# Pediatric age estimation from thoracic and abdominal CT scout views using deep learning

**DOI:** 10.1038/s41598-023-29296-3

**Published:** 2023-02-08

**Authors:** Aydin Demircioğlu, Kai Nassenstein, Lale Umutlu

**Affiliations:** Institute of Diagnostic and Interventional Radiology and Neuroradiology, University Hospital Essen, University of Duisburg-Essen, 45147 Essen, Germany

**Keywords:** Machine learning, Paediatric research

## Abstract

Age assessment is regularly used in clinical routine by pediatric endocrinologists to determine the physical development or maturity of children and adolescents. Our study investigates whether age assessment can be performed using CT scout views from thoracic and abdominal CT scans using a deep neural network. Hence, we retrospectively collected 1949 CT scout views from pediatric patients (acquired between January 2013 and December 2018) to train a deep neural network to predict the chronological age from CT scout views. The network was then evaluated on an independent test set of 502 CT scout views (acquired between January 2019 and July 2020). The trained model showed a mean absolute error of 1.18 ± 1.14 years on the test data set. A one-sided t-test to determine whether the difference between the predicted and actual chronological age was less than 2.0 years was statistically highly significant (*p* < 0.001). In addition, the correlation coefficient was very high (R = 0.97). In conclusion, the chronological age of pediatric patients can be assessed with high accuracy from CT scout views using a deep neural network.

## Introduction

Pediatric age assessment is part of clinical practice and has applications in many different contexts. In forensics, the aim is to identify deceased individuals^1^, whereas, in medicolegal applications, the goal is to determine whether a minor with an unknown date of birth is of legal age^2^.

The most often use-case is in endocrinology, where age assessment is used to determine whether a growth disorder is present in a pediatric patient^3^. In clinical practice, two well-known methods by Greulich-Pyle and Tanner-Whitehouse are used^4,5^. Both are based on a radiograph of the non-dominant hand, in which the skeletal maturity of the hand bones is compared with reference radiographs of minors of known chronological age. While these methods show high accuracy, a disadvantage of such atlas methods is that they are very time-consuming. In addition, they still exhibit great intra- and interrater variability^6^. Automating this task would reduce the time effort while simultaneously leading to a certain standardization, decreasing the intra- and interrater variability. Accordingly, deep learning methods^7^ based on artificial neural networks have been employed to automate age assessment^8^. A distinct advantage to other machine learning methods is that deep learning is able to extract problem-relevant features automatically^9^. Thus, they can reach human-like accuracy in specialized tasks^10^.

Depending on the use case, methods based on different parts of the body and different imaging modalities have been proposed. For example, for forensic use, the most common way to assess the age is by analysis of a radiograph of the teeth^11^. This method can be used regardless of the patient’s age, and a corresponding atlas method was developed^12,13^. In medico-legal context, where the goal is to be fully certain that a patient cannot be younger than a certain age, methods based on the ossification of the clavicula have been put forward, which can be performed in radiographs and CT scans^2,14,15^.

**Figure 1:**
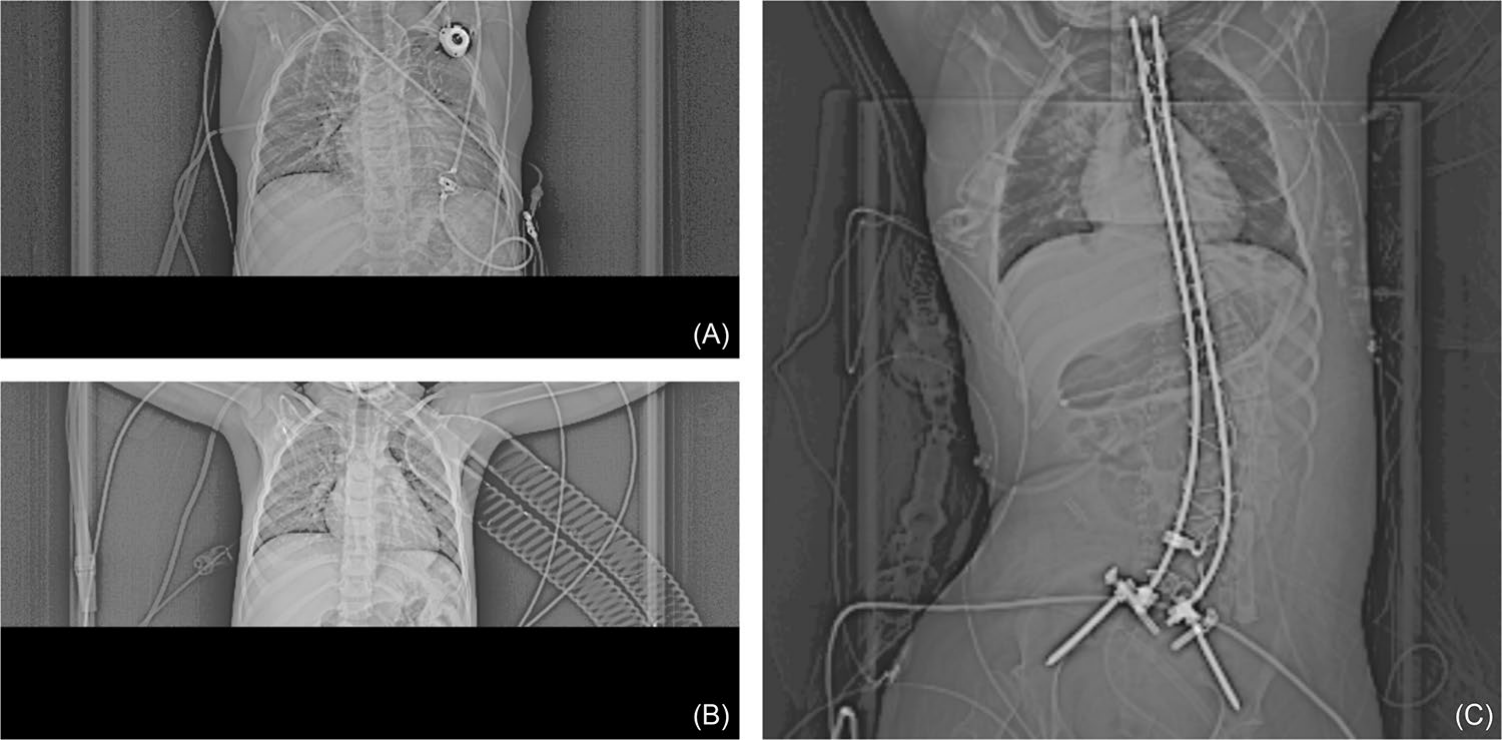
Preprocessed CT scout views for two patients. (**A**) The CT scout view for a CT thorax examination of a male patient (5.3 years) (**B**) The CT scout view for a CT thorax examination of a male patient (2.8 years) (**C**) The CT scout view of a CT abdomen examination of a female patient (19.5 years).

In clinical practice, CT scans of the thorax and abdomen are frequently performed. For planning such examinations, overview images with low radiation exposure, so-called CT scout views are acquired beforehand (Fig. 1). Despite their auxiliary role, CT scout views, have a value of their own^16,17^. As CT scouts are routinely stored in the picture and communication system (PACS), they can be easily accessed. In contrast to CT scans, they consist of only a single image; thus their handling is much easier from a technical viewpoint. Since CT scout views display a large area of the body and are commonly acquired in routine clinical imaging, the question arises if CT scout views can be used to assess the chronological age of pediatric patients.

This work aimed to use deep learning methods to determine the chronological age of pediatric patients using CT scout views from thoracic and abdomen CTs. For this, we collected corresponding CT scout views from our university hospital between 2013 and 2020. We trained several different network architectures and loss functions and tested their predictive performance in terms of mean average error (MAE). Since, in general, networks have various hyperparameters that need to be optimally chosen for high prediction performance, we employed a hyperparameter optimization framework for training.

## Results

### Patient collective

**Figure 2:**
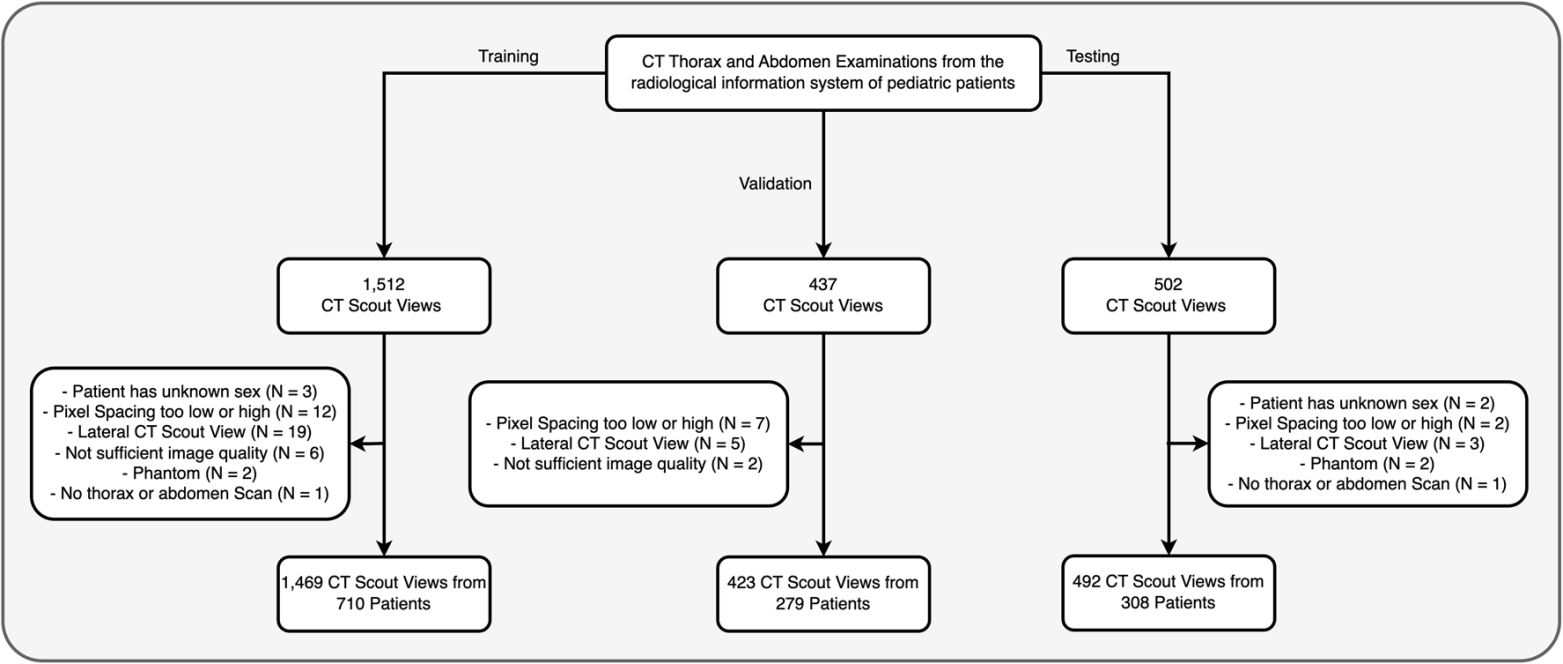
Patient flowcharts with inclusion and exclusion criteria.

**Figure 3:**
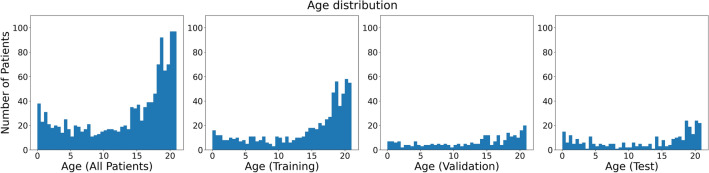
Histogram of the age of the patients. From left to right: Of all patients, of patients in the training set (N = 710), of patients in the validation set (N = 279), of patients in the test set (N = 308).

Altogether, 1,469 CT scout views were collected for training, 423 for validation, and 492 for testing (Fig. 2). The mean age of all patients was 13.5 ± 6.6 years (range: 0.0–21.0 years), with 590 females and 707 males (Table 1 and Fig. 3).

**Table 1:** Demographics of the patient collective. Age is reported in years.

	All	Training	Validation	Test
Female	45% (590/1297)	46% (330/710)	41% (113/279)	48% (147/308)
Male	55% (707/1297)	54% (380/710)	59% (166/279)	52% (161/308)
Age	13.5 ± 6.6 (N = 1297)	14.2 ± 6.3 (N = 710)	12.8 ± 6.5 (N = 279)	12.5 ± 7.2 (N = 308)

### Evaluation of the validation set

**Figure 4:**
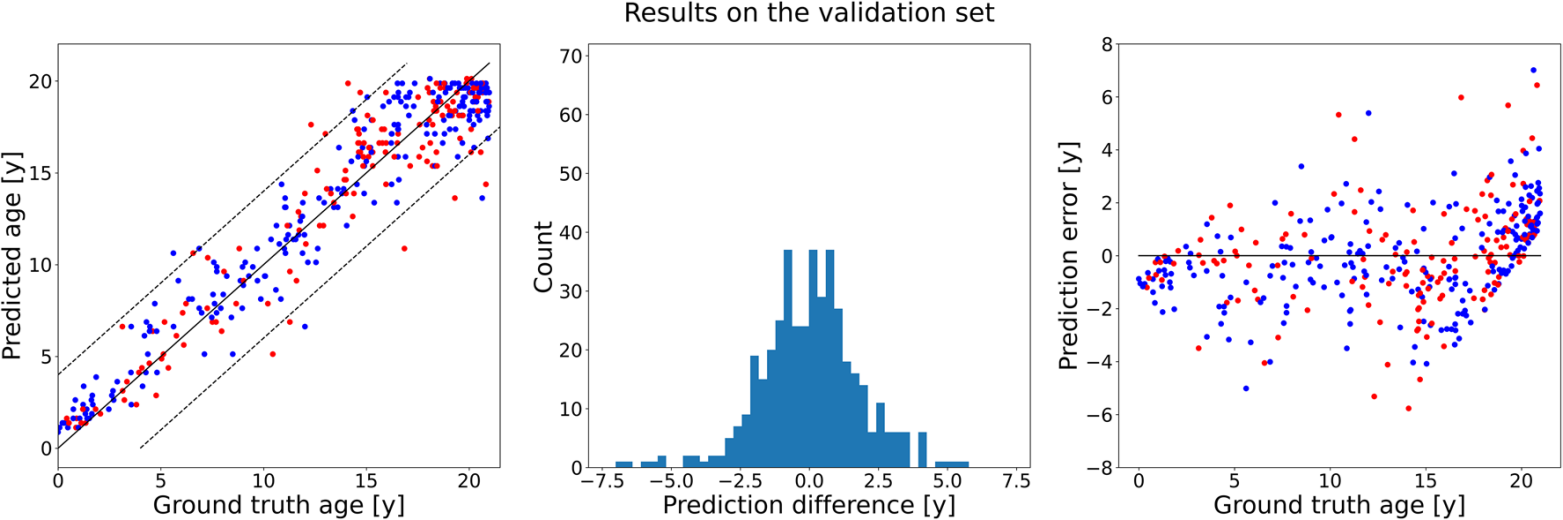
Results of the optimized network trained on the training set and evaluated on the validation set. **Left**: Scatter plot of the ground truth ages (*x*-axis) against predicted ages (*y*-axis). The dashed lines mark the limits at ± 4 years. Red dots correspond to female patients, blue dots to male patients. **Middle**: A histogram of the prediction errors. **Right**: Scatter plot of the ground truth age (*x*-axis) against the prediction errors (*y*-axis). Red dots correspond to female patients, blue dots to male patients.

Hyperparameter optimization showed that the network performed best with the adaptive mean-residue (AMR) loss^18^ and the DenseNet-121 network^19^ as backbone, and obtained an MAE of 1.37 ± 1.17 years (Fig. 4). The other methods performed slightly worse, with the network using L1 loss performing close (1.39 ± 1.44 years), and the CORAL and CORN methods with lower performance (Table 2). The loss curves of the best-performing model for each method can be found in the supplementary materials.

**Table 2:** Performance of the optimized networks in the validation set. Since AMR performed best in terms of MAE, it was chosen as final model.

Network loss function	MAE on validation set [years]	Error (> 2 years)	Error (> 4 years)
AMR	1.37 ± 1.17	23.2% (98/423)	4.5% (19/423)
CORAL	1.55 ± 1.54	29.3% (124/423)	6.6% (28/423)
CORN	1.48 ± 1.46	22.2% (94/423)	6.6% (28/423)
L1	1.39 ± 1.44	24.6% (104/423)	5.7% (24/423)

**Figure 5:**
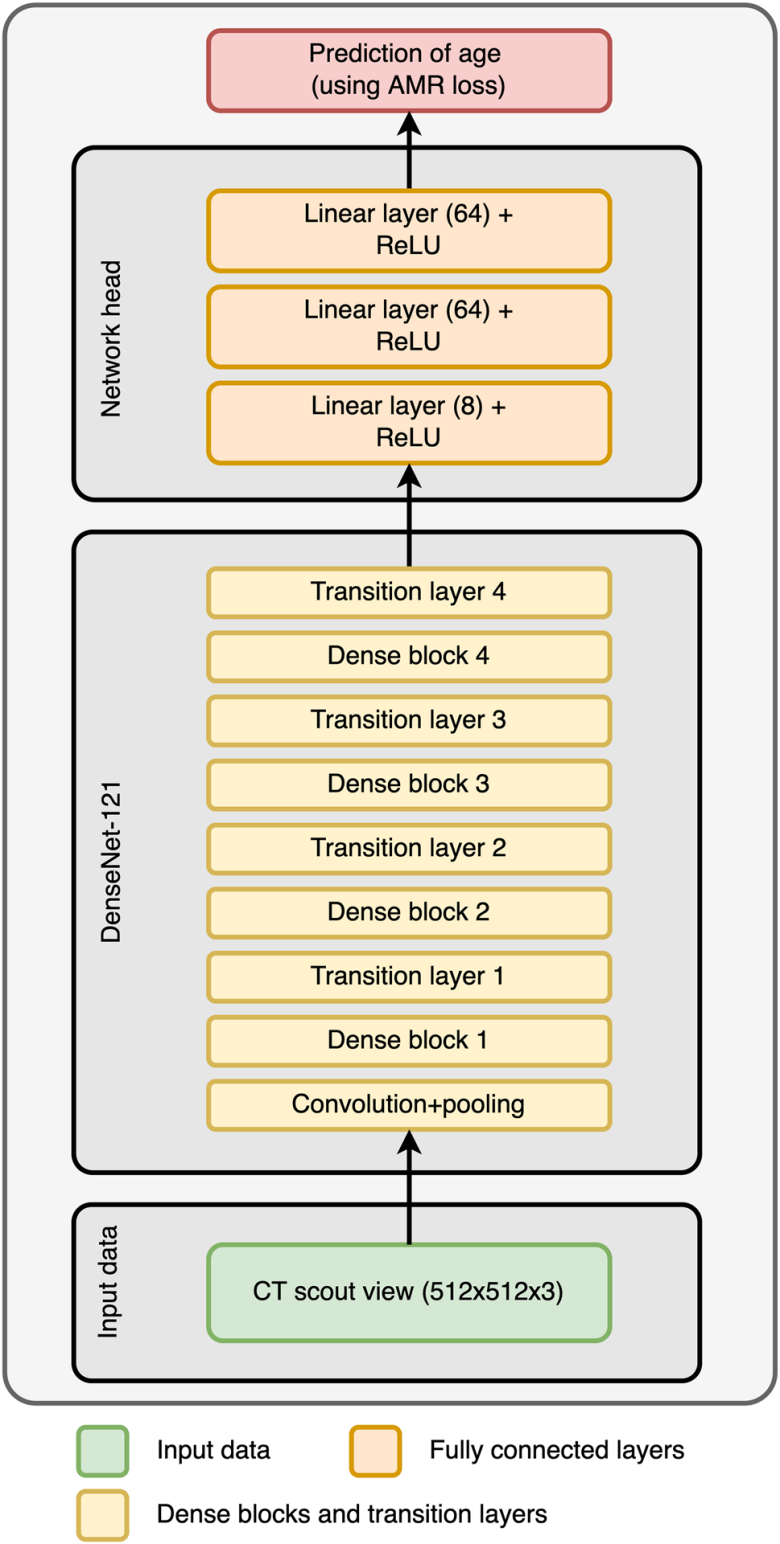
The optimal network architecture found by the hyperparameter tuning. The network was initialized with weights trained on the ImageNet data set. No part of the network was frozen during training, yielding around 7.0 million tunable parameters.

Therefore, the AMR method was selected as the best-performing model. Its loss parameters were chosen to be K = 4 and λ_2_ = 0.013. This optimized network comprised head layers with sizes [8, 64, 64] (Fig. 5). The weights were pretrained on the ImageNet data^20^, with no layer frozen, containing around 7.0 million trainable parameters. The learning rate was set to 2.5*10^-4^, and was multiplied by 0.6 every 15 epochs. Learning was stopped early at epoch 42, as no more progress could be seen in the test set.

### Evaluation of the test set

**Figure 6:**
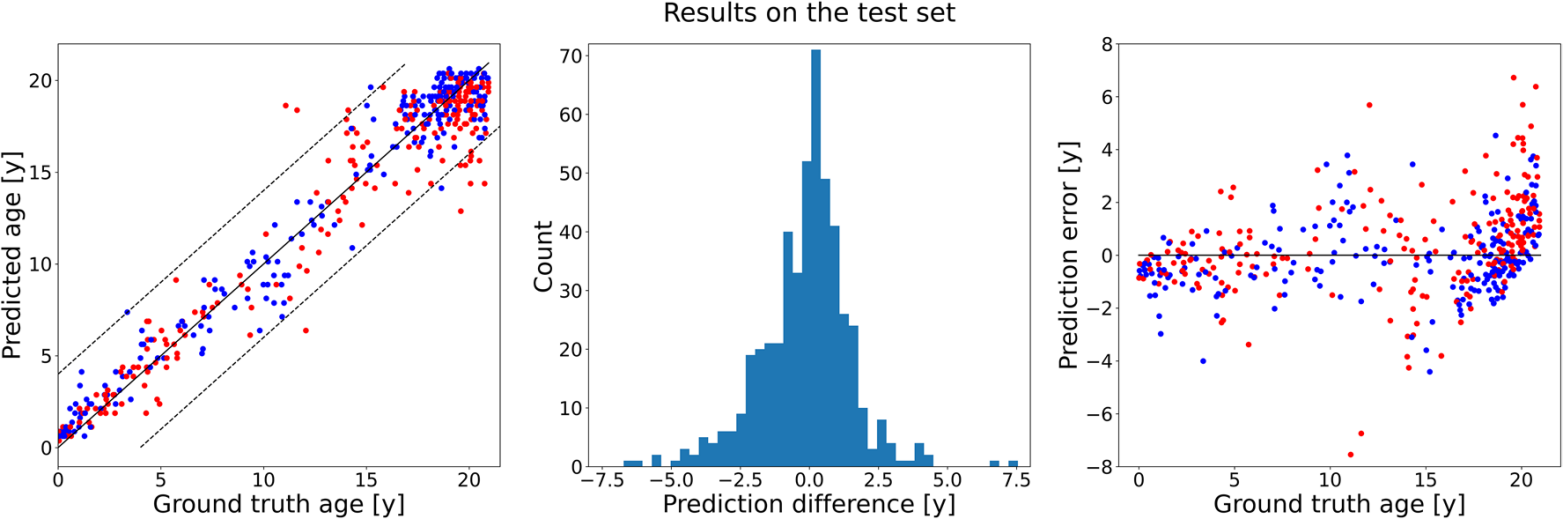
Results of the optimized network retrained on all data, i.e., the training set as well as the validation set, and evaluated on the independent test set. **Left**: Scatter plot of the ground truth ages (*x*-axis) against predicted ages (*y*-axis). The dashed lines mark the limits at ± 4 years. Red dots correspond to female patients, blue dots to male patients. **Middle**: A histogram of the prediction errors. **Right**: Scatter plot of the ground truth age (*x*-axis) against the prediction errors (*y*-axis). Red dots correspond to female patients, blue dots to male patients.

**Figure 7:**
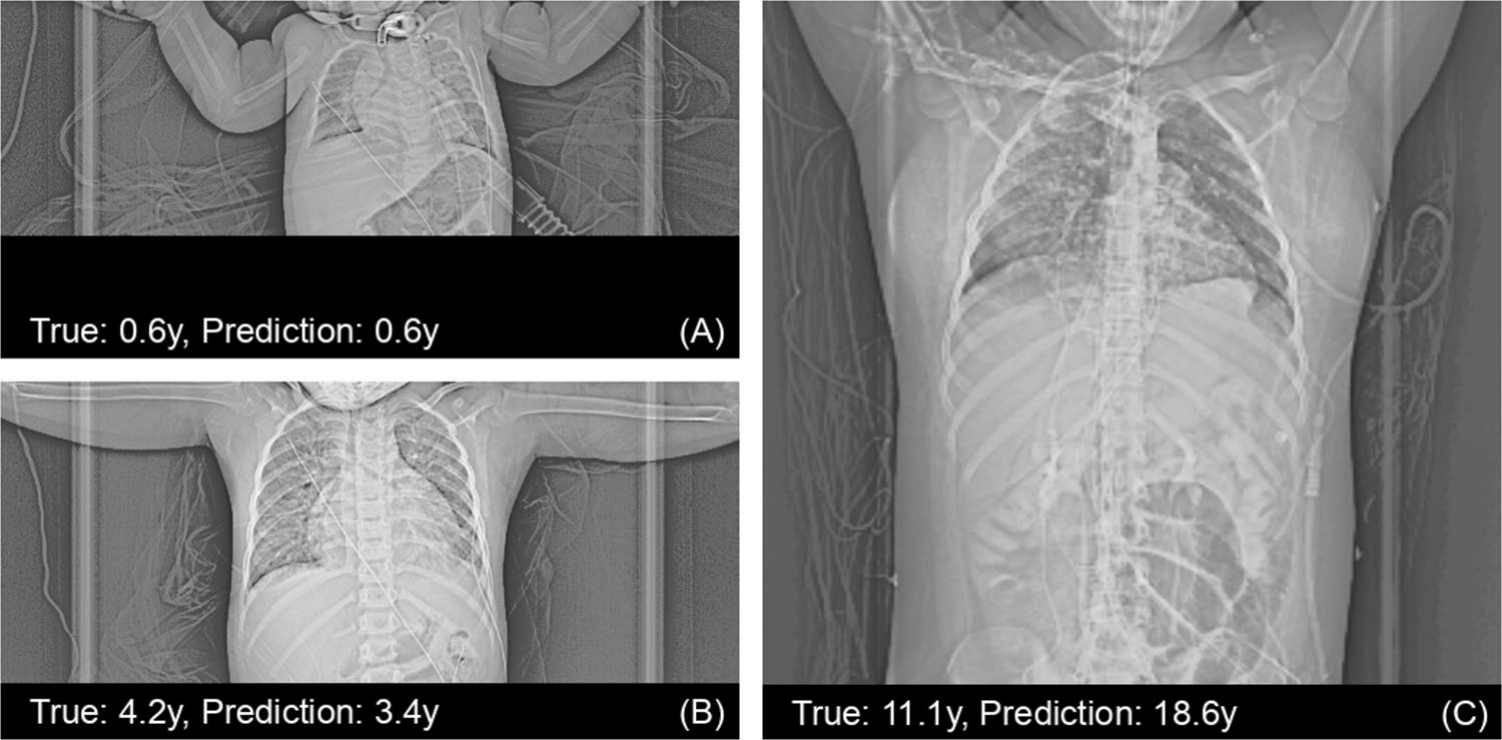
Three scout views from the test set, where the prediction error of the optimized network was (**A**) near zero, (**B**) near the median error, and (**C**) worst over all scout views. Scout views were cropped to increase visibility. Note that in (**C**) a pulmonary ground glass opacity can be seen, possibly due to COVID-19.

Using the hyperparameters with highest performance on the validation set, the network was retrained using all available training data to exploit the available information as much as possible, i.e., the training data as well as the validation data was used. The number of epochs was kept the same at 42 epochs. The trained model was then evaluated on the independent test data. The accuracy of this model was 1.18 ± 1.14 years (Fig. 6). A small increase in performance could be seen in accuracy when compared with the performance on the validation set (0.19 years in MAE). Three exemplary scout views where the prediction error was near zero, near the median error, and was worst are shown in Fig. 7.

A one-sided t-test was used to test whether the mean absolute error was smaller than 2.0 years. The test was highly significant (*p* < 0.001). The error in age exceeded the limit of 2.0 years in 17.7% (87/492) of all cases, while in 3.0% (15/492) the error exceeded 4.0 years (Fig. 6). Pearson correlation showed that the correlation of the predicted and true outcomes for age were excellent (R = 0.97).

### Significance maps

**Figure 8:**
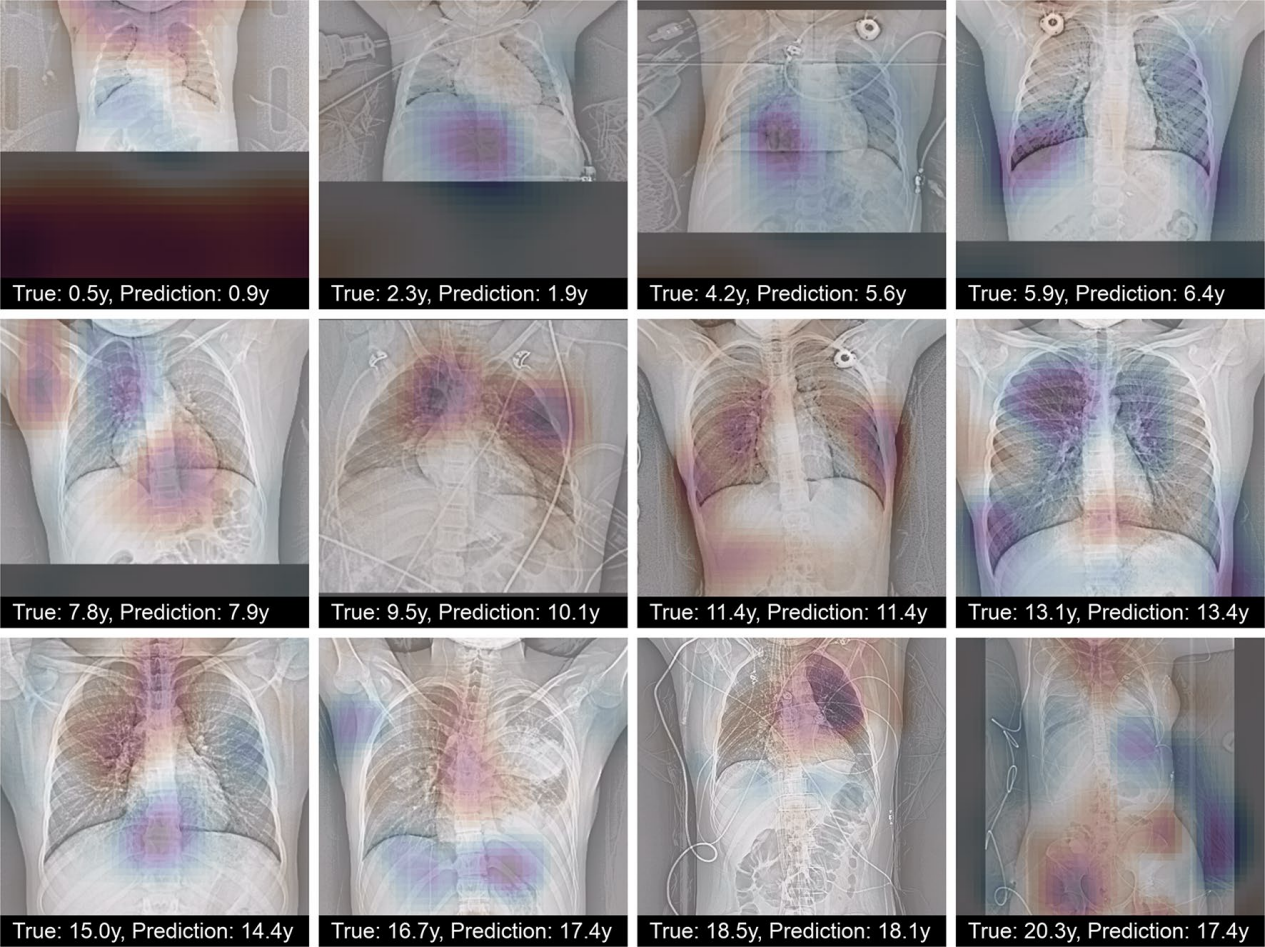
Occlusion maps for the optimized network for selected scout views from the test set. Scout views were selected evenly within the age range. Regions with darker color contributed more to age regression. The occlusion of red regions increased the prediction of the network compared to the prediction using the whole image, and the occlusion of blue regions decreased it.

To understand to which parts the network paid the most attention, occlusion maps^21^ were computed using a window of size 48 × 48 and a stride of 3. These indicated that the cervical and thoracic spines, and the area of the aortic arch were important for prediction (Fig. 8).

## Discussion

Age assessment in pediatric patients using the maturity of the hand bones is a well-known technique that has been automated^8,22^. While age assessment on other body parts have already been proposed and are in use in practice, the automation of these using machine learning and deep learning techniques is not yet widespread.

Our results show that the automated assessment of age is possible using CT scout views of thoracic and abdominal CT scans. The mean average error achieved was 1.18 ± 1.14 years, which can be regarded as rather accurate. Still, age assessment using hand radiographs is more accurate, reaching typically error levels of around 4–5 month^23^, though recently Gong et al. improved the accuracy substantially and obtained a mean average error of less than 2 months^22^. A key difference between both approaches lies in the data used for training. While hand radiographs are often obtained solely for age assessment, CT scout views are only auxiliary in nature and only obtained as a byproduct of CT scans. Therefore, our data is much more heterogeneous when compared to hand radiographs, where no pathology is generally expected. Only very few scout views were excluded (less than 3%), and the two main reasons for exclusion were that a phantom was scanned or the scout view was not properly acquired. It is very likely that with a more homogenous data set, higher accuracies could be reached. In addition, hand bone data sets are commonly larger as the examination takes place more frequently; for example, the hand bone data set used in the RSNA challenge contains over 12,000 radiographs^8^. It is well known that larger data sets lead to increased performance for deep neural networks. A similar effect was seen for the network used in this study, too, as the network trained on all training data (train and validation data set) showed a better performance than the network trained only on the training data set.

Pediatric age assessment on other body parts have been explored and are used for different use cases but in general are not as accurate as the assessment based on hand bones. Sauvegrain et al. developed a method to assess skeletal age in elbow radiographs^24^, which can be used mainly for patients in puberty age and is thus complementary to hand bone age. Similarly, radiographs of the pelvis can be used for age assessment, where a high correlation between the ossification of the iliac crest has been demonstrated for patients of age between 10 and 30 years^25^. For knees, an atlas was prepared by Pyle and Hoerr^26^. Janczyk et al. considered low-dose, whole-body dual-energy x-ray scans of patients up to 19 years and obtained an MAE of 1.3 years, which is comparable to our results^27^.

Age assessment was also performed on different image modalities. A promising alternative to radiographs is MR imaging, as MRI scans do not involve potentially harmful exposure to radiation. Accordingly, methods have been developed for age assessment based on MRI of hand bones^28^, the iliac crest^29^ and knees^30^.

Although these methods work considerably well, the long exposure times of MRI are a common drawback, especially for younger patients. Methods based on ultrasound were also proposed^31^, but suffer partially from the same drawback. Fewer methods are based on CT imaging, for instance in forensic context maturity of the clavicula is often used in adolescents and young adults^32^. Recently, age assessment based on the first rib was proposed using multi-slice CT^33^.

One reason that CT imaging is not more frequently used despite its higher resolution is the much higher radiation exposure involved, which makes applicability in pediatric context questionable. Our proposed method is no exception. Although CT scout views are acquired in low-dose, and thus are not as harmful as full CT scans, they are only auxiliary in nature and thus are always acquired in combination with a CT scan involving much higher exposure. Therefore, our method is only applicable if a CT scan has to be acquired because of other reasons and is only complimentary in nature.

We restricted our study to pediatric age assessment, which is routinely applied in clinical practice to detect growth disorders. However, estimation of age in older patients can be useful as well, for example to relate the health of a patient to age-expected health. Using chest *X*-rays of the very large NIH Chest *X*-ray8 dataset, Karargyris et al. trained a deep network to regress age in adult patients^34^. Their model showed an accuracy of 67% of prediction of age within 4 years. Similarly, Yang et al. trained a deep model on chest *X*-rays of healthy subjects and obtained an MAE of 2.1 years^35^ They employed group-summarized activation maps and identified the cervical, thoracic spines, first ribs, aortic arch and the heart region as important for prediction. Our model seems to confirm this observation since in our visualization these areas had also the highest influence on the prediction.

Age estimation can also help assess status or predict disease onset. Franke et al. used Bayesian methods to regress adult brain age on T1-weighted MRI scans of the brain^36^. They trained their model on healthy subjects only, yielding an MAE of 5.0 years, but showed that the model had a much larger estimation error in patients suffering from early Alzheimer's disease (AD). Subsequently, Gaser et al. exploited the model to predict whether a mild cognitive impairment would develop into AD within 3 years^37^. Their model showed 81% accuracy. Recently, Hepp et al. trained a three-dimensional convolutional neural network on a German national cohort study^38^. Their model was more accurate and achieved a MAE of 3.2 ± 2.5 years; their methods were also able to quantify the uncertainty of the prediction, which was 2.9 ± 0.6 years. It can be expected that this more sophisticated model could lead to better predictions in the future. Whereas these studies were performed only on adults, Shi et al. presented a model for predicting brain age from T2-weighted fetal MRI scans^39^. They were able to show that their model, with an MAE of 0.767 weeks, was able to predict the presence of multiple prenatal brain diseases with high accuracy.

The results in this study show high accuracy, yet some limitations apply. First and foremost, the study is based on a single site with CT scanners from a single vendor. Therefore, validating the results on other sites, ideally with an ethnically diverse population, is necessary. A further limitation concerns the network: we used four common off-the-shelf architectures with different loss functions. Still, some overfitting was visible during training (see Figure S1 in the Supplementary material)—even though we have used several techniques to reduce overfitting. Namely, we employed pretraining with weights trained on ImageNet, freezing different layers (which reduces the overall number of parameters), early stopping, data augmentations, dropout, and the AdamW optimizer (which also contains a weight decay term). Curiously, the performance on the test set was better than on the validation set, which indicates less severe overfitting. Still, a more custom-tailored and smaller network could overfit less and could possibly, with more refined data, yield prediction errors below one year. Our approach underlines that neural networks can work very well even with heterogenous data and without expert skills in network architectures or tuning.

In conclusion, the chronological age of pediatric patients can be assessed with high accuracy from CT scout views using a deep neural network, which can be easily adopted in clinical routine.

## Materials and methods

Ethical approval for this retrospective study was granted by the local ethics committee (Ethics Commission of the Medical Faculty of the University of Duisburg-Essen; registry number 21–10,069-BO). Written and informed consent was waived by the Ethics Commission of the Medical Faculty of the University of Duisburg-Essen because of the retrospective nature. The study was performed in accordance with all relevant guidelines and regulations.

### Patients

The study population was collected by querying our radiological information system for thoracic and abdominal CT examinations of pediatric patients aged < 21 years which comprise a CT scout view in anterior–posterior direction. For training of the model, CT scout views of 1512 patients examined between January 2013 and December 2017 were collected anonymously (Fig. 2). Similarly, a validation set was created using 437 CT scout views of patients examined between January 2018 and December 2018. Finally, a test set of 502 examinations was gathered by querying all pediatric patients examined between January 2019 and June 2020. Since patients could have several examinations at different times, care was taken to ensure that each patient was only part of exactly one of the three data sets in order to avoid bias. No random selection was used during the collection, that is all available examinations were included into the data sets as long as the patients’ age was below 21 years. Exclusion criteria were: A pixel spacing of the CT scout view of 0 mm (indicating a broken CT scout view) or more than 2 mm, or missing information on the sex of the patient (the latter two indicating either a phantom or an acquisition error). All CT scout views were reviewed by a radiologist and scans were additionally excluded if they either comprised either of the following: (1) CT scout view in lateral direction; (2) body parts than thorax and abdomen; (3) phantom scan; (4) insufficient image quality. In the end, 1,469 CT scout views of 710 patients for training, 423 CT scout views of 279 patients for validation and finally 492 CT scout views of 308 patients for an independent test (Fig. 2) were used.

### CT scout view acquisition

All CT scout views were acquired in inspiration in anterior–posterior direction on several Siemens CT scanners (Siemens Healthineers, Erlangen, Germany) (Table 3). Tube voltage varied between 80 and 140 kV and tube currents between 20 and 100 mA. Overall, the scout views were acquired on 23 different scanners.

**Table 3:** CT scanners used to acquire the CT scout views. Scanners with less than 50 examinations were gathered into the “Other” group and contained 18 scanners (for example, SOMATOM Emotion 6, Emotion 16, Sensation 4, Sensation 16, etc.).

	All (N = 2384)	Train (N = 1469)	Validation (N = 423)	Test (N = 492)	Tube voltage	Tube current
SOMATOM definition flash	855	678	98	79	120 kV	20 mA
SOMATOM force	702	210	220	272	120 kV	35 mA
SOMATOM definition AS +	467	336	56	75	120 kV	36 mA
SOMATOM definition AS	142	63	36	43	120 kV	35 mA
Volume zoom	113	113	0	0	120 kV	100 mA
Other	105	69	13	23	80–140 kV	20–60 mA

### Preprocessing

All CT scout views were first retrieved in anonymized fashion and then converted to 8-bit PNGs for better handling. To remove a few outlier pixels, HU values below − 256 and above 1024 were clipped; each scan was then rescaled linearly to the range 0–255. Contrast limited adaptive histogram equalization (CLAHE)^40^ was then applied with two different parameters [clip limit = 64, grid tile size = (1,1); clipLimit = 32, grid tile size = (2,2)]; these two and the original image were then merged to obtain an RGB image. Images had a fixed resolution of 512 × 512 pixels (Fig. 1).

### Neural network

Four different methods were tested to predict age from CT scout views. The first method consists of a simple regression; in other words, the network directly predicts the age and uses the L1 loss to measure its errors. We call this method accordingly as L1. The other three methods treat the regression problem as an ordinal classification, that is, as a series of binary tasks. Each binary task answers whether the patient is older than a certain age or not. The combination of these answers then leads to a prediction. The first of these methods is CORAL (consistent rank logits), which introduces a consistency condition to ensure that the binary tasks do not contradict each other; this leads to higher coherence and, therefore, better predictions. CORN (conditional ordinal regression) is a direct improvement upon CORAL; it accounts for conditional probability distributions and solves a few technical issues. Finally, the adaptive mean-residue loss (AMR) method was applied, which adds a residual loss to reduce the probability of predictions outside the top-*K* classes closest to the true age.

In addition, we tested whether adding DICOM-tags (like exposure time or used kilovoltage peak) can improve over the network with L1-loss. More details and the results can be found in the Supplementary material.

### Hyperparameter tuning

A separate hyperparameter tuning search for the best network was performed for each method to allow direct comparison. Each search consisted of finding an appropriate network architecture (selected from ResNet-18, Res-Net 34, ResNet-50, and DenseNet-121) to serve as the backbone and the layer sizes of a fully connected head (with three layers) of the network^41^. Furthermore, the tuning optimized whether to use weights trained with the ImageNet data set^20^ and how much of the network layers to freeze. All networks were trained using the AdamW optimizer^42^; the learning rate and its scheduling by a step-based scheduler were also considered hyperparameters and optimized. Early stopping was employed to prevent overfitting. Finally, the image size was also subjected to tuning, where either the original size of the ImageNet images (224 × 224 pixels) or the full resolution of the scout views (512 × 512 pixels) could be selected.

Hyperparameters were tuned using the “Optuna” framework based on Tree Parzen Estimators^43^. The network was developed using Python 3.8 and Pytorch 1.13. All details of the training procedure are described in full in the Supplemental material.

### Sample size estimation and statistical analysis

A one-sided t-test was conducted to test the hypothesis whether the age can be predicted with a mean average error less than 2.0 years. For sample size calculations, using as standard deviation of the training set, 5.9 years, and a significance level of alpha = 0.05 as well as a power of 0.9, the required sample size for evaluation was determined to be 93.

### Ethical approval

All procedures performed in studies involving human participants were in accordance with the ethical standards of the Institutional Research Committee and with the 1964 Declaration of Helsinki and its later amendments or comparable ethical standards.

## Supplementary Information

Supplementary Information.

## Data Availability

The datasets generated during and/or analysed during the current study are available from the corresponding author on reasonable request. For reproducibility, the code for training the neural network and evaluation will be made available on GitHub (https://github.com/aydindemircioglu/scout.view.age).
