# Supplementary material for: Pediatric age estimation from thoracic and abdominal CT scout views using deep learning
Source: Sci Rep. 2023 Feb 8;13:2274. doi: 10.1038/s41598-023-29296-3 (PMC9907877; doi:10.1038/s41598-023-29296-3)
Supplement: Supplementary file 1 — Supplementary Information. [file 41598_2023_29296_MOESM1_ESM.pdf]

Supplemental Material

Pediatric Age Estimation from Thoracic and Abdominal CT Scout Views using Deep Learning

Neural Network architecture

All networks considered during hyperparameter search consisted of a backbone (selected from ResNet-18, ResNet-34, ResNet-50, and DenseNet-121) and a fully connected head. The head replaced the final classification layers of the backbone and comprised three fully connected layers. Between the backbone and the head, a dropout layer (with fixed dropout of 0.1) was applied. The loss of the network was optimized using the AdamW optimizer (with default values betas = (0.9, 0.999) and a weight decay of 0.01). The batch size was chosen as large as feasible, which was 32. The maximum number of epochs was fixed at 100. Early stopping was used to prevent overfitting and training was stopped when the decrease in the validation loss was less than 0.05 over 20 epochs.

For the three methods using ordinal classification, a direct conversion of age to a label would not allow for prediction accuracy below one year; accordingly, ages were converted into classes by multiplying by 4 to allow for a higher precision. This conversion was reversed after prediction, so as not to affect the MAE by this conversion.

Augmentations

Neural networks benefit strongly from small image transformations called augmentations which change the appearance of an image without destroying the information contained. For example, rotating a CT scout view will generally result in a different appearance but does not change the

patient's age. Therefore, only small rotations (between -10 and 10 degrees), small changes in brightness and contrast (up to 10%), and random paddings (up to either 32 pixels for images that are 224x224 pixels or 64 pixels for images that are 512x512 pixels) with subsequent cropping to the original image size were applied. The latter transformation amounts to randomly shifting the image in horizontal and vertical directions. In addition, since most CT scout views are centered in the upper half of the image, padding was applied anisotropically, i.e., padding was not applied to the bottom of the image since this cropping could cut off a large part of the patient's body.

Hyperparameter Optimization

The neural network architecture and its training depend on several hyperparameters one must choose appropriately. To optimize all these parameters, "Optuna", a hyperparameter optimization library based on Tree Parzen Estimators, was chosen for efficient tuning[1]. In detail, the following parameters were optimized by Optuna:

- The choice of network backbone (ResNet-18, ResNet-34, ResNet-50, DenseNet-112)
- The size of the three fully connected layers of the head (each between 4, 8, 16, …, 1024)
- The image size (either the original resolution of 512x512 pixels or downscaled to the size of the ImageNet data set, 224x224 pixels)
- The freezing of pretrained layers (between -1 and 4; -1 = use random weights, 0 = use pretrained weights, 1..4 = use pretrained weights and freeze parts of the network)
- The learning rate (log-uniform in the range 0.1 and 1e-6)
- The learning rate schedule (gamma in 0.1, 0.2, …, 1.0 and step size in 15, 16, …, 30).

For the AMR loss, two additional parameters were tuned: the top-K number (K in 3, 4, … 10) and the λ_2 parameter (in 0.0, 0.01, 0.02, … 0.2). The λ_1 parameter was fixed to 0.2, as suggested in the original study.

Scheduling was performed by multiplying the learning rate with the chosen gamma after a given number of epochs (called step-size scheduling). Finally, the freeze parameter was optimized, which determined how many parameters of the backbone were trainable or fixed during training. For this, the layers of each network architecture were roughly divided into four parts. In more detail (using the naming conventions of the respective studies by He et al.[2] and Huang et al.[3]):

- For the DenseNet-121: Freeze = 1 froze convolutional+pooling layers and dense block 1 and transition layer 1. Freeze = 2 froze in addition dense block 2 and transition layer 2. Freeze = 3 froze in addition dense block 3 and transition layer 3. Freeze = 4 froze all layers.
- For the ResNets: Freeze = 1 froze convolutional+pooling layers and conv2_x. Freeze = 2 froze in addition conv3_x. Freeze = 3 froze in addition conv4_x. Freeze = 4 froze all layers.

Weights of fully connected layers were initialized using the method by He et al.[4] and were always trainable. A full overview of the amount of trainable and non-trainable parameters can be found in Table S1.

**Image size**	**Freeze level**	**Parameter**	**DenseNet-121**	**ResNet-18**	**ResNet-34**	**Res-Net-50**
224	0	Trainable	6,953,856	11,176,512	21,284,672	23,508,032
		Non-trainable	0	0	0	0
	1	Trainable	6,576,000	11,019,008	21,053,184	23,282,688
		Non-trainable	377,856	157,504	231,488	225,344
	2	Trainable	5,524,224	10,493,440	19,936,768	22,063,104
		Non-trainable	1,429,632	683,072	1,347,904	1,444,928
	3	Trainable	2,160,128	8,393,728	13,114,368	14,964,736
		Non-trainable	4,793,728	2,782,784	8,170,304	8,543,296
	4	Trainable	0	0	0	0

		Non-trainable	6,953,856	11,176,512	21,284,672	23,508,032
512	0	Trainable	6,953,856	11,176,512	21,284,672	23,508,032
		Non-trainable	0	0	0	0
	1	Trainable	6,576,000	11,019,008	21,053,184	23,282,688
		Non-trainable	377,856	157,504	231,488	225,344
	2	Trainable	5,524,224	10,493,440	19,936,768	22,063,104
		Non-trainable	1,429,632	683,072	1,347,904	1,444,928
	3	Trainable	2,160,128	8,393,728	13,114,368	14,964,736
		Non-trainable	4,793,728	2,782,784	8,170,304	8,543,296
	4	Trainable	0	0	0	0
		Non-trainable	6,953,856	11,176,512	21,284,672	23,508,032

Table S1: Parameter count for the backbones of the network architectures. The freeze level refers to the layers that were frozen during training. A freeze level of 0 will not freeze any layer, while a freeze level of 4 will freeze all weights of the backbone; only the network head with fully connected layers will be trainable in this case.

The total rounds of parameter sets to be searched by Optuna were fixed to 100. In each optimization round, Optuna selected the hyperparameters of the network, which were then trained on the training data set and evaluated on the validation set by computing the MAE. After optimization, the hyperparameters of the best-performing model in terms of MAE was chosen as the final model.

After the best network structure was determined, the network was trained using both training and validation data sets. This is because, in general, neural networks benefit from more data. The training was conducted as many epochs as during the training of the best model. The retrained model was considered to be the final model. Its performance was then evaluated on the independent test data set. This final evaluation took only place once to avoid introducing any bias by repeatedly optimizing for the test set, which would lead to severe overfitting.

Loss curves

To judge the overall quality of the training of the best model and whether overfitting occurred, the loss curves of the best-performing models were plotted (Fig. S1).

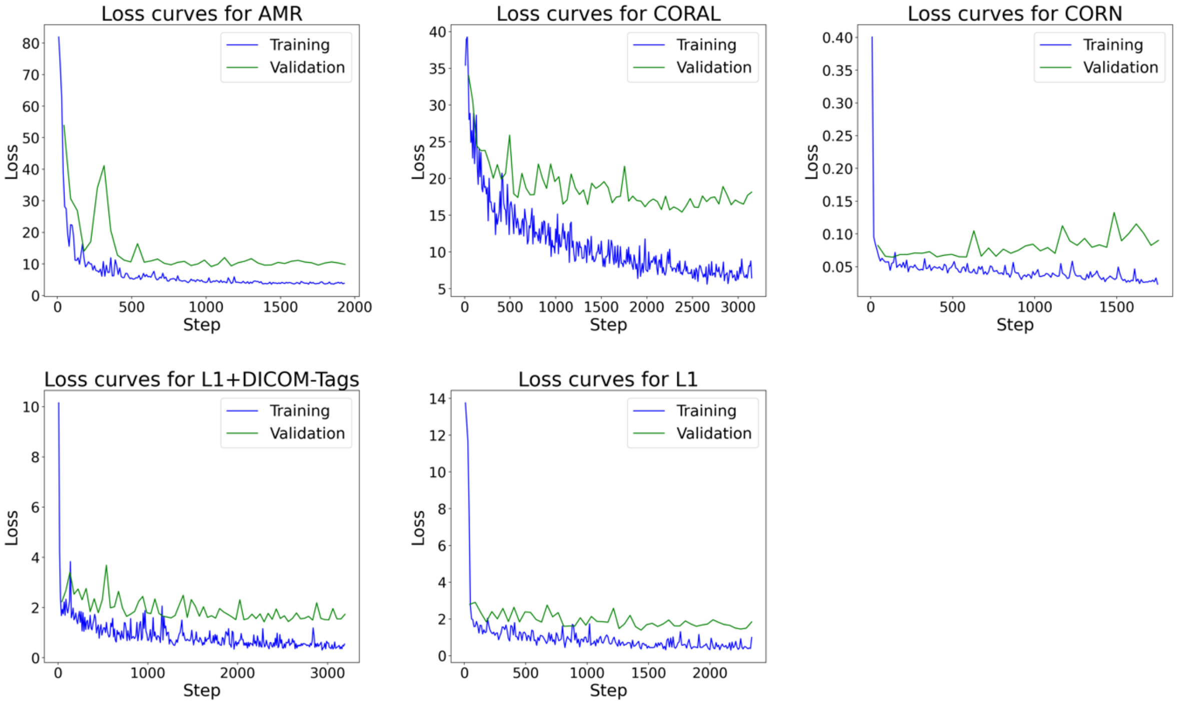

Figure S1 Loss curves for the best-performing model. Note that since the methods use different loss functions, their absolute value cannot be compared directly (except for the two methods using L1 loss).

Benefit of DICOM tags

Since the acquisition of scout views depends on several imaging parameters like exposure time and kilovoltage peak, we tested whether such information could help in improving the network predictions. For this, we extracted the following DICOM tags from each scout view: kilovoltage

peak (KVP), x-ray tube current, exposure (in mAs), the computed tomography dose index (CTDIVol), the maximum of the pixel spacing in both directions. In addition, the sex of the person was extracted from the DICOM tags as it is known to be an important factor in child and adolescence maturity and growth. Missing DICOM tags affected mainly the CTDIVol tag (N = 170 in the training set, N = 7 and N = 1 in the test and validation set resp.) and only marginally the Exposure tag (N = 5 in the training and N = 2 in the test set). All missing DICOM tags were replaced by the mean of the corresponding tag in the training set, i.e., CTDI was replaced by 0.11 while Exposure was replaced with 184.9.

These tags were then input into a small network with three fully connected layers and ReLU activations. The sizes of these layers were considered to be hyperparameters and were subjected to tuning. The output of this network was then concatenated to the encoded feature vector from the backbone and then processed with several fully connected layers. Apart from this change, the very same procedure was performed, i.e. the same hyperparameter tuning was used.

The optimized network used the ResNet-50 as backbone, and used the CT scout views at full resolution; it used pretrained weights, but all layers were trainable. The fully connected layers processing the DICOM tags had sizes of [4, 4]; the head layers processing the concatenated feature vector had a size of [8, 1024, 1024] while the learning rate was 1.5e-4 and was multiplied by 0.9 every 22 steps.

This network showed an MAE of 1.43 ± 1.45 years on the validation set, which was slightly higher than the 1.39 ± 1.44 years of the same procedure without DICOM tags. Therefore, it seemed that adding DICOM tags to the network did not improve predictive performance.

Software

The neural network was developed using the Python 3.8, Pytorch 1.13[5]. For reproducibility, the code for training the neural network and evaluation will be made available on GitHub (https://github.com/aydindemircioglu/scout.view.age).

References

1. Akiba, T., Sano, S., Yanase, T., Ohta, T. & Koyama, M. Optuna: A Next-generation Hyperparameter Optimization Framework. *ArXiv190710902 Cs Stat* (2019).
2. He, K., Zhang, X., Ren, S. & Sun, J. Deep Residual Learning for Image Recognition. in *2016 IEEE Conference on Computer Vision and Pattern Recognition (CVPR)* 770–778 (2016). doi:10.1109/CVPR.2016.90.
3. Huang, G., Liu, Z., van der Maaten, L. & Weinberger, K. Q. Densely Connected Convolutional Networks. *ArXiv160806993 Cs* (2016).
4. He, K., Zhang, X., Ren, S. & Sun, J. Delving Deep into Rectifiers: Surpassing Human-Level Performance on ImageNet Classification. in 1026–1034 (2015).
5. Paszke, A. *et al.* PyTorch: An Imperative Style, High-Performance Deep Learning Library. *ArXiv191201703 Cs Stat* (2019).
